# Alertness can be improved by an interaction between orienting attention and alerting attention in schizophrenia

**DOI:** 10.1186/1744-9081-7-24

**Published:** 2011-07-05

**Authors:** Isabelle Amado, Juan Lupiañez, Marion Chirio, Steffen Landgraf, Dominique Willard, JP Jean-Pierre Olié, Marie Odile Krebs

**Affiliations:** 1 INSERM, Physiopathologie des Maladies Psychiatriques, U894-7; Centre de Psychiatrie et Neurosciences Paris, France; 2 University Paris Descartes, Faculty of Medicine Paris Descartes, Service Hospitalo-Universitaire, Hôpital Sainte-Anne, Paris, France; 3 University of Granada, Faculty of Psychology, Department of Experimental Psychology, Granada, Spain; 4 Service de Psychiatrie, Hôpital Saint Jacques, Nantes, France; 5 University of Humboldt, Institute of Psychology, Berlin, Germany

## Abstract

**Background:**

Attention is impaired in schizophrenia. Early attention components include orienting and alerting, as well as executive control networks. Previous studies have shown mainly executive control deficits, while few of them found orienting and alerting abnormalities. Here we explore the different attentive networks, their modulation and interactions in patients with schizophrenia.

**Methods:**

Twenty-one schizophrenic patients (DSMIV), compared to 21 controls, performed a modified version of the Attention Network Task, in which an orienting paradigm (with valid, invalid and no cues) was combined with a flanker task (congruent/incongruent) and an alerting signal (tone/no tone), to assess orienting, executive control and alerting networks independently.

**Results:**

Patients showed an abnormal alerting effect and slower overall reaction time compared to controls. Moreover, there was an interaction between orienting and alerting: patients are helped more than controls by the alerting signal in a valid orientation to solve the incongruent condition.

**Conclusion:**

These results suggest that patients with schizophrenia have altered alerting abilities. However, the orienting and alerting cues interact to improve their attention performance in the resolution of conflict, creating possibilities for cognitive remediation strategies.

## 1. Introduction

Attention encompasses different functions, which work together in everyday life and are dissociable from perception and action: orientation of attention, triggering an alert state, and resolving response conflicts. The orienting network selectively allocates attention to a potentially relevant area of the visual field, enhancing perceptual processing. The alerting network prepares for action by means of a change in internal state. This preparation can be triggered when a visual or auditory warning signal is presented prior to a target. Executive control involves planning, decision making, error detection, giving novel responses, or overcoming habitual actions [1]. The executive control of attention is a top-down process that generates "descending feed back signals that bias sensory inputs in favour of information that is behaviourally relevant" [2]. It is also involved in the resolution of conflict between competing information and regulates activity in other brain networks involved in thought and emotion [3].

The distinction between orienting, alerting, and executive control is also evident on the neuronal level [4]: The orienting neural network allocates attention to relevant areas of the environment and involves the posterior parietal lobe, the superior colliculus, and the thalamus [5]. Local infusion of scopolamine into the posterior parietal cortex in rhesus monkeys during a covert orienting task showed a dose dependent increase in reaction time (RT) and decrease in performance accuracy. This indicates a major role of the posterior parietal cortex in orienting, with a pivotal cholinergic influence in the modulation of orientation [6]. The alerting network comprises right thalamic, frontal and parietal regions [4] and neuroimaging studies using PET and fMRI during reaction time tasks with and without warning, also showed an involvement of the reticular formation [7]. Sturm and Wilmes (2001)[7] provided neuropsychological evidence that alerting is subserved by right hemispheric regions with parieto-fronto-thalamic interactions. Alerting is influenced by the cortical distribution of the brain's norepinephrin system that arises from the locus coeruleus [8]. PET studies during the performance of the Stroop color word task in healthy controls showed that the executive network is subserved by the anterior areas of the frontal cortex, i.e. the anterior cingulate cortex [9] and the lateral prefrontal cortex [10], which are target areas of the ventral tegmental dopamine system [11]. During a visual discrimination task with fMRI requiring exploratory eye movements, Tsunoda et al [12] showed that the number of fixations correlated with decreased gray matter in right frontal and parietal structures. Using a diffusion tensor imaging technique, Buchsbaum et al. (1998) [13] found lower diffusion anisotropy in the white matter of the prefrontal cortex in patients with schizophrenia compared to controls. Impairments of attentional processes in schizophrenia are heterogeneous; difficulties in disengaging visuo-spatial attention have been observed in chronic never-medicated schizophrenics [14], acute naïve patients [15] and relatives of patients [16], as well as in patients treated with typical [17,18] or atypical antipsychotics [19]. Abnormal reactivity to warning signals has been described as a deficit in the maintenance of an alert state in naïve [20] or treated patients [21]. In addition, schizophrenics show a marked sensitivity to interference in the Stroop [22] and flanker task [23], or in oculomotor paradigms such as antisaccade tasks [24], which are considered to represent executive functioning. Moreover, using cerebral imaging techniques, hypoactivity in the anterior cingulate cortex has been visualised during a conflict monitoring task [25].

The Attentional Networks Test (ANT), is a paradigm designed to examine attentional network efficiencies in orienting attention, alerting, and executive control [26,4]. The task is based on the combination of (i) a cued reaction time (RT) task and (ii) a flanker paradigm which evaluates the ability to solve conflicts. ANT studies in patients with schizophrenia have previously yielded inconclusive results: 1) An overall slower RT [27-29]. 2) A deficit in executive control was found in a vast majority of studies [27,28,30,31], while only one study found no executive deficit [29]. 3) A deficit in orienting, found in some [27] but not all studies [29]. 4) Reduced alertness, which was correlated to illness duration and medication levels. Tensor diffusion brain imaging indicated that the reduction in alertness was correlated to a smaller right cingulum bundle volume [29]. However, in many studies reporting an executive deficit in patients, these differences might have been maximized by discrepancies in group matching. Some studies were carried out with populations matched for age [28], or comparing populations with pre-existing differences in executive function as detected by the Wisconsin Card sorting test [29], other studies were performed with differences for patients versus controls in number of years of study [31]. Only one study had a strict comparison between patients and controls for IQ level and years of education [27].

The variability of the ANT results in schizophrenia could result from confounding factors due to the type of population itself (IQ, age, treatment), but also to the intrinsic properties of the ANT task. Indeed, MacLeod et al. (2010) [32] highlighted different psychometric properties of the ANT that could complicate the results found: the relative dependence of the three different networks measurements, the low reliability of the alerting and orienting score compared to the executive control scores, and the high intra-individual variability observed in control studies. Lastly, some components of attention could be trait-like (e.g executive control), or state-like (alerting or orienting) explaining the variance structure of the ANT scores.

Callejas et al. [33,34] used a modified version of the ANT paradigm to dissociate orienting, alerting, and executive control and to study their interactions. The version of ANT used by Callejas et al. [33,34] is remarkable in two important aspects: 1) The presence/absence of the non-valid trials: in this version, orienting is measured using a *non-predictive *cue, with 50% valid vs. 50% invalid cues. The ANT includes only 100% valid cues. 2) Alerting is assessed with an *auditory*alerting cue whereas a *visuo-spatial*cue is used in the ANT. The non-predictive cue allows the activation of an exogenous orienting system (with the automatic capture of attention by a non-informative visual stimulus). In the original ANT, the 100% valid cue condition induced an impure, mixed exogenous and endogenous orientation of attention (exogenous attention raised by the cue presentation, and endogenous attention raised by the always predictive meaning of the cue). Also, in Callejas et al.'s [33] version, the warning sound stimulus fundamentally differs from the orienting visual stimuli, whereas in the ANT, the alerting and orienting networks are activated by the same four types of visual cues. These two differences make the modified Callejas et al version of ANT [33]suitable for a) measuring the function of each attentional network independently, and b) studying interactions between these networks. In healthy subjects, Callejas et al. [34] found that the alerting network influences the executive control network by inhibiting its functioning, which is consistent with Posner's proposal (5). Moreover, the alerting network influences the orienting network by speeding up the orienting process. Fuentes et al. [35] showed, using this ANT version in healthy controls, that orienting to the target location in advance enhanced target processing speed and reduced conflict. The conjunction of a mixed positive action on orienting and conflict, suggests that alerting improves rather than accelerates the orienting effect. Ishigami and Klein (2010) [36] stated that the Callejas' version of ANT "permits the researcher to examine the interaction among the attentional networks with confidence". Concerning the robustness of the attention network scores when examining the repeatability of the ANT and ANT-I tests (Callejas ' version), these authors argued that "despite the learning effect, the two tests provided robust index of each attention network", but "overall the reliability of the network scores was found to be greater with the ANT-I than the ANT".

Investigation of the different attentional networks in schizophrenia and the modulation of their effects on each other are of crucial importance. On the one hand, the interactions of attentional networks occur constantly in everyday life, suggesting that deficits would have a wide impact. On the other hand, disentangling specific deficits in schizophrenia could provide the basis for new cognitive remediation techniques to enhance the attentional abilities of patients with schizophrenia. Gooding et al.(2006) [28], using the ANT, attempted to analyze the interactions between different attention networks. They showed that both patients with schizophrenia and controls were most efficient in resolving conflict when they were alerted and when their orientation was towards the attended spatial position. Neither Gooding et al. [28] nor Wang et al. [27]' found correlations between the attentional networks. In Gooding et al. (2006)[28] this was due to the confounding factor that alerting and orienting cues were both presented in the visual modality. Fan et al. (2009)[37] recently provided a revised version of the ANT with the aim of characterizing attentional network interactions in healthy volunteers. The authors manipulated the length of the cue-target interval and cue validity (with a no cue, spatial cue and temporal cue condition) and found that orienting to the target location before the alerting stimulus enhanced target processing and reduced conflict. Nevertheless, Gooding et al. (2006)[28] suggested using the ANT version of Callejas et al. (2004,2005)[33,34], which allows the independent measurement of the three attentional networks and their interactions. This modified task could, thus, reduce the heterogeneity of the results found in schizophrenia using the original ANT paradigm and highlight the influence of the orienting network on executive control in patients with schizophrenia.

ANT and its modified version are supposed to explore attention interactions between alerting, orienting and executive control in a relatively independent way. This notion might be modulated, since it was found "that there was some lack of independence among the networks in both tests [36]". Moreover, cued paradigms including mixed block design such as the ANT task not only induced processes of activation (in generating an alerting effect for example) but also proactive response inhibition processes [38].

The purpose of our study is to explore (1) whether orienting, alerting, or executive control are altered in patients with schizophrenia, (2) how the three networks interact in patients, Both groups of participants were strictly matched for age, sex and IQ level to minimize the differences that might exist in general aptitudes. In line with the majority of studies, we expected executive control of attention to be impaired in patients, but due to our strict matching this difference could not be found, whereas impairment in alerting or orienting could be evidenced. Therefore, we administered for the first time the Callejas' version of ANT (2004)[32] to stable outpatients with schizophrenia and healthy controls.

## 2. Methods

### 2.1. Participants

Characteristics of the participants are summarized in table 1. Twenty-one outpatients with chronic schizophrenia (SZ), and 21 healthy controls (C) (15 males in both SZ and C) participated in the study. Study procedures were described before participants decided to take part in the study and signed their written informed consent. SZ and C received 30 Euros for their participation. Study procedures were approved by the local Ethical Committee (CCPPRB- Pitié-Salpétriére Hospital, Paris). Participants were not instructed to restrain their cigarette consumption before the assessment. However, they were not allowed to smoke during the session test.

**Table 1:** Demographic and clinical characteristics: values are expressed as means and standard deviations

	SZ	C	P
Gender	15M/6F	15M/6F	
Age (years)	31(8)	30(9)	0.76
Years of study	13(2)	13(2)	0.99
WAIS-R Global	101(14)	105(15)	0.4
WAIS-R Perform	95(11)	103(20)	0.2
WAIS-R Verbal	103(18)	106(17)	0.7
Handedness	17D/4M	18D/3M	0.5

^D: Dextral; S: Sinistral; M: mixed lateralization^

For all subjects, exclusion criteria were: a patent neurological disease, a history of head trauma, substance abuse or dependence. All participants were assessed with the Diagnostic interview for Genetic Studies, DIGS-III [39], the NSS (neurological soft signs) scale [40], and the WAIS-R [41].

SZ (DSM-IV criteria)[42] were recruited from the Ambulatory Center of the Parisian 15th arrondissement and the University Department of Psychiatry at Sainte-Anne Hospital, Paris, France. Mean duration of the disease was 9 (± 6) years. Clinical evaluations were conducted with the PANSS (Positive and Negative Syndrome Scale, [43]. PANSS scores at the time of neuropsychological testing were: Total score: mean = 59, SD = 13, Positive subscore: mean = 12, SD = 4, Negative subscore: mean = 17, SD = 6. All patients had received stable monotherapy with atypical antipsychotics (risperidone < 4 mg, clozapine, olanzapine, amisulpride < 600 mg) for at least three months (mean duration: 29 (24) months) prior to investigation (equivalent chlorpromazine dosage: 388 mg (207 mg). No other medication was allowed.

C were recruited from our Clinical Research Center, excluding members of the department, subjects that had ever had a DSM-IV axis one disorder, and subjects with a family history of a psychiatric disorder up to the 2nd degree. None of C had ever received any psychotropic medication.

### 2.2. Attentional task (figure 1)

**Figure 1:**
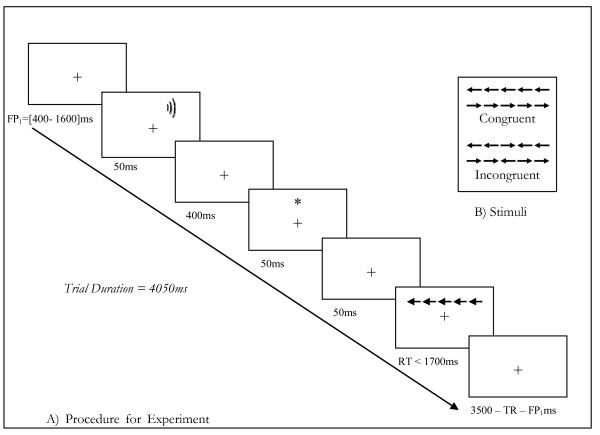
**Sequence of events appearing on each trial in the modified version of ANT experiment**. Part A shows the actual sequence of events during the ANT task in our study. Part B shows examples of the target display in the congruent and incongruent condition. Stimuli: The stimulus used for the orienting signal was an asterisk presented at the same location as the target (2.9° of visual angle above or below the fixation point). For the alerting signal, a 2000 Hz and 50 ms sound was used. Lastly, the target display was made up by a target arrow that could point either to the left or to the right and four flankers that could be just plain black lines or arrows pointing either left or right. The length of the arrows was 0.55° and they were 0.06° away from each other. Description of the task: In half of the trials, 50 ms before the target an auditory alerting signal was presented (fig1). After a 400 inter-stimulus-interval (ISI), an orienting cue was presented on 2/3 of the trials above or below the fixation point for 50 ms. After another 50 ms ISI the target and flankers were presented either at the same or the opposite location than the previous orienting signal for 1700 ms, or until the participant gave a response. Then the fixation point that had been presented during the whole trial was kept for a variable duration dependent on the duration of the initial fixation point and on the reaction time of the subject so that every trial was same duration (4050 ms). No screen was presented between trials. Consequently, participants did not know when a trial had finished and the next one was to begin providing uncertainty about the appearance of the signals and increasing their informative value.

#### 2.2.1. Apparatus

Programming and presentation of stimuli was performed with an Intel-Pentium-4 computer with a 17" color screen monitor running E-Prime software.

#### 2.2.2. Procedure

Participants were seated 53 cm in front of the computer screen and instructed to respond to the target stimulus (direction of the central arrow), by pressing one of two possible keyboard keys using their right or left index finger, depending on the side of the answer. Feedback regarding accuracy was given during practice trials but not during experimental trials.

#### 2.2.3. Task Design (figure 1)

The experiment had a multivariate mixed design including two levels for the Alerting Signal (presence/absence of a sound) × three levels for the Orienting Cue (No Cue/Valid/Invalid) × two levels for the Executive Control (congruent/incongruent, see Figure 1) × 2 Groups (SZ/C). Altogether, there were 12 different conditions (see Figure 1). The practice block (24 trials) proceeded the six experimental blocks of 48 trials each. The whole task included 24 trials per condition with a pseudo-random presentation within each block. Overall, the duration of the experiment took nearly 20 minutes; patients had a pause between each block and decided to start the next block by pressing the space bar.

### 2.3. Statistical analyses

Trials with RTs longer than 2000 ms were eliminated and the ratio for incorrect responses in each trial was reported. Median RTs were analyzed with a 2 (Alerting Signal) × 2 (Orienting Cue) × 2 (Congruent/Incongruent) × 2 (Group) mixed ANOVA. Effect size was evaluated with eta^2 ^proportion of the total variance that attributed to an effect. Different indexes were established: an alerting effect (RT difference between the alerting cue and no alerting cue conditions), an orienting effect (RT difference between the valid and invalid conditions) and a conflict effect (RT difference between the congruent and incongruent conditions) (33); In each group, one way ANOVAs were used for comparison regarding gender and smoking on these indexes. Pearson correlation coefficients were used to explore associations between clinical, IQ, and index variables. In order to illustrate more precisely the link between symptoms, IQ and performance we examined very contrasted groups for clinical symptoms and IQ, subdividing the SZ group into two categories (high vs. low score for PANSS and IQ, cut-off: 3^rd ^quartile of the distribution), and we calculated the means of the effect in each category.

## 3. Results

### 3.1. Clinical and demographic data

There were more men than women in SZ and C. However, there was no significant difference between SZ and C for sex ratio, age, years of study, or IQ (see table 1).

### 3.2. RT analysis (table 2)

**Table 2:** Median RT(ms) and percentage of errors (for each experimental condition, for Patients (SZ),) and healthy Controls (C)

		No Alerting Cue						With Alerting Cue					
		
		invalid		No Cue		Valid		Invalid		No Cue		Valid	
SZ	Congruent	646	0,90%	656	0,40%	615	0,20%	617	0,40%	589	0,40%	584	0,70%
	Incongruent	754	4,10%	756	3,30%	716	1,30%	738	4,60%	703	2,80%	650	0,90%

C	Congruent	568	0,40%	584	0,80%	540	0,20%	561	0,60%	533	0,20%	514	0,20%
	Incongruent	690	3,10%	662	1,10%	624	1,10%	679	4,00%	640	2,70%	612	1,60%

RTs exceeding 2000 ms were very few (0.17%) with no difference between patients and controls. The main effects for the three attentional systems were significant. Trials with an auditory alerting signal were faster than those without one [F(1, 40) = 27.36; p < .001;eta^2 ^= 0.41]. Participants were faster when oriented with valid cues compared to invalid cues [F(1,40) = 175.31; p < .001; eta^2 ^= 0.81]. Finally, participants were faster in congruent trials compared to incongruent ones [F(1,40) = 204.12 p < .001; eta^2 ^= 0.84]. Considering performance between the two groups, the main effect was significant, [F(1,40) = 5.98; p = .019; eta^2 ^= 0.13]. SZ were 66.5 ms slower than C.

Importantly, we observed a significant alerting × group interaction, [F (1, 40) = 5.41; p = .025; eta^2 ^= 0.12] with SZ (mean = 36 ms) showing a greater sensitivity to the auditory alerting cue than C (mean = 14 ms). Finally, the four-way interaction of orienting × alerting × control × group was statistically significant, [F (1,40) = 6.21; p = .017; eta^2 ^= 0.13] and other interactions with group were non-significant (p > 0.25).

**Figure 2:**
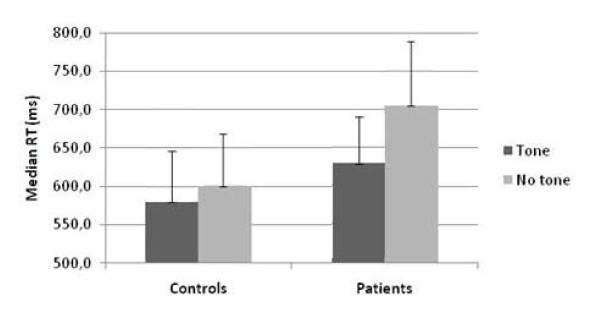
**comparison of reaction times between patients and controls in incongruent valid conditions in the absence or presence of an auditory alerting signal**. Deviations are indicated by use of median absolute deviation.

Partial ANOVAs were conducted to disentangle the four-way interaction. Separate analyses were conducted on the four congruent/incongruent × orienting conditions. In the incongruent valid cue condition the 2 (Alerting Signal) × 2 (Group) mixed ANOVA showed a significant alerting × group interaction [F (1, 40) = 13.13; p = .001], indicating that SZ benefited more than controls from the alerting effect in the incongruent condition when their attention was validly oriented (alerting index in SZ: 66 ms in SZ in C:14 ms) (Figure 2). In others conditions (incongruent- invalid; congruent-valid; congruent-invalid) this interaction was non-significant (p > 0.18).

### 3.4. Accuracy analysis

The ANOVA performed on the percentage of incorrect responses revealed a main effect for the Orienting Cue, [F(1, 40) = 18.41; p < .001], and the Congruent/incongruent factor, [F(1, 40) = 14.56 p < .001]. The Orienting Cue × Congruent/incongruent interaction was significant, [F(1, 40) = 19.13; p < .0001]. The orienting effect was higher in incongruent than in congruent conditions. In other words, when participants are not spatially oriented, accuracy is worse for the incongruent trials. No group effects were found (Figure 2).

### 3.5. Correlations between performance and demographical and clinical data

In SZ, in the absence of an alerting signal (no tone condition), the conflict effect was negatively correlated with WAIS-R performance (r = -.46; p = 0.041). Furthermore, SZ with low WAIS-R-performance scores (RT = 750 ms; SE = 39 ms) had longer RTs than those with high WAIS-R performance scores (RT = 714 ms; SE = 32 ms) in the incongruent condition, whereas there was no difference in the congruent condition.

Similarly, in C the conflict effect was negatively correlated with years of study (r = -.46; p = 0.041).

## 4. Discussion

In accordance with our assumption that the ANT version of Callejas et al., [33,34] allows differentiation of the three attentional networks and their interactions, we obtained the following main experimental results:

1) SZ exhibited slower overall RTs than C,

2) SZ displayed a greater alerting effect compared to C.

3) In SZ only, an interaction was found between alerting and orienting.

In line with studies using the ANT [27-29,31], SZ were slower than C. In our study, the reported effects were independent of this general slowing, and remained significant even when we used proportional RT as a dependent variable in a supplementary analysis (median RT per condition divided by overall median RT, data not shown).

Chronic stabilized outpatients showed a greater alerting effect using this version of ANT than C. Previous studies with ANT have not reported this result and indeed have found unchanged [27,28,31] or reduced alerting efficiency [29]. This could be due to differences in the modality of the alerting stimulus. In Callejas et al.'s version of the ANT [33], the alerting stimulus is auditory, the orienting stimulus is visual. In contrast, alerting and orienting cues are ipsimodal (visual) in prior ANT versions. SZ were slower than C in all no-tone conditions, suggesting that their reactivity was relatively decreased. The sound cue seemed to enhance alertness in patients but their overall RTs remained longer than those seen in C. In healthy subjects, Frassinetti et al. (2002)[44] reported that an auditory stimulus can enhance the detection of masked visual flashes. Recently, Noesselt et al., (2008)[45] tested an auditory enhancement of perceptual sensitivity to visual blinks in a two-alternative choice paradigm. They found that a crossmodal visuo-auditory cue significantly enhances detection ability, as if this enhancement increases the salience of the visual event. Our results show that patients are sensitive to this increase in target salience, helping them to reduce their RTs. Ishigami and Klein (2010)[36] suggested that the auditory modality of the Callejas' ANT version [33,34] might generate alertness more automatically than the visual modality of the ANT could do. The more automatic alert state induced in this task could help patients more efficiently, opening fruitful ways to enhance performance in schizophrenia that could be used in cognitive remediation techniques.

Our results clearly demonstrate a greater sensitivity in patients to auditory alerting stimuli compared to controls. However, although reaction times when alerted provided a substantial positive effect in patients, it was not enough for them to reach the performance of healthy subjects. In other words, even if patients improved their alertness ability with the tone, they still showed lower reaction times in every condition with an auditory cue. Nestor et al., (2007)[29] found that a reduction in visual alerting cues correlated with a reduced volume of the cingulum bundle. These results together with our findings may demonstrate a failure of alertness in schizophrenia. Recently, in an experimental study concerning the induction of a psychotic-like state in healthy subjects, Daumann et al., 2009 [46], using auditory and visual cueing stimuli, found a reduction in alertness after a bolus injection of Dymethyltriptamine, a hallucinogenic drug. Thiel and Fink, (2007)[47] provided evidence in healthy subjects for modality specific correlates of visual and auditory alertness in posterior parietal and frontal brain areas. However, a supramodal region, the right superior temporal gyrus, was commonly involved in visual and auditory alertness. This brain region is involved in the behavioural relevance of warning cues [48], and "its activation is capable of breaking ongoing activity and optimizing responses to following target(s)" [47]. Abnormal superior temporal gyrus volumes have been found in schizophrenia [49].

Nevertheless, our attempt to superimpose analyses of the effect of valid or invalid trials, or congruent or incongruent trials provided comparable orienting or conflict effects in SZ and controls. This result contradicts those studies that suggest an executive control deficit [27,28,30,31]. Chronicity, hospitalization, non-stabilization, IQ, and educational level could be confounding factors. In our study, patients and controls were strictly matched in years of education and IQ. Neuropsychological studies comparing schizophrenic patients and controls most often reported less educational achievement in patients, reflecting the impact of the disease [27]. The strict comparison of attentional performance by equalizing the confounding IQ and education features is an original aspect of our study. It provides a more stringent comparison of the three attentional networks, highlighting the power of the difference found in the alerting network. It can also explain the absence of an executive control deficit found in our patients, by a possible underestimation of the neuropsychological differences between patients and controls. In our study educational level was negatively correlated with the conflict effect in controls. Moreover, we found convergent arguments supporting the relationship between IQ and executive control: 1) In SZ the conflict effect in the no-tone condition was negatively correlated with WAIS-R performance. 2) RTs in the incongruent condition differed depending on IQ performance.

Lastly, we found an interaction between alerting and orienting in SZ, especially for the incongruent condition. In incongruent trials, in the valid orientation condition, when there is no auditory alerting signal, SZ benefited less than controls from the valid orientation; this tendency being reversed with the presence of the auditory alerting cue. In controls, in contrast, the absence or presence of a valid cue has an equivalent effect whatever the presence of an auditory alerting signal. One should ask whether the effect of a valid spatial cue was reduced in patients owing to the presence of invalid trials. In the present paradigm, valid trials indeed represent 50% of the non-neutral trials, as compared to 100% in the ANT studies. The auditory alerting cue would then compensate for this decreased validity effect. This last point brings confirmation to the facts that 1) the addition of invalid trials is a main change relative to previous studies 2) This study was aimed at clarifying interactions between the orienting and alerting network. If we consider the accuracy analyses, it seems that in both groups invalid and incongruent conditions led to the worst performance. Thus, in patients, the adjunction of a correct orientation and a warning tone enhanced their ability especially in difficult conditions e.g with incongruent stimuli. This finding is in line with the interaction between orienting and alerting in healthy subjects observed by Fuentes et al., (2008)[35] using the Callejas version of the ANT [33,34], and Fan et al. (2009)[37] with the modified ANT. Patients seem to extract more benefit than controls from this adjunction of cues. This observation could bring valuable tools to enhance attention in schizophrenia, with a putative application in cognitive remediation strategies.

Regarding cognitive remediation in schizophrenia, meta-analyses have shown no direct benefit of training techniques of attention [50,51]. Although not improving attentional measures, these techniques could positively influence executive dimensions and optimize working memory, response speed, or visual scanning. Accordingly, improving the capacity to solve executive conflicts by reinforcing an alert state in patients or helping them to focus their attention could possibly improve efficiency. The results found in this study with the Callejas et al. ' version of the ANT [33] provide a way to investigate attentional networks in schizophrenia. The combination of orienting and alerting strategies in attentional tasks assessing conflict resolution could be adopted routinely in integrative programs of cognitive remediation therapy.

The results found in this study address the question of the specificity of these alterations, to determine whether the attentional processing style observed here is likely to represent an illness feature, i.e. a consequence of the expression of the illness, or a measure of some of the aetiological factors of schizophrenia, e.g a cognitive marker of the disease. If the latter, this abnormality could be studied as a putative endophenotype of schizophrenia. Of course, the present design did not allow us to address this question but it would be very useful to study the performance of relatives of patients with schizophrenia. Also, an exploration of patients with prodromal symptoms or schizotype personalities might be useful to see if early alterations of the attentional networks could represent possible stage markers of the disease.

Several limitations of this work can be found. First, the restricted sample size could limit the power of our analysis. However, the strict inclusion criteria used to select the participants in our study tend to increase the homogeneity of the two groups. Further studies are warranted using this version of the ANT with a more extended group of subjects. Second, the participants in the two groups have high general aptitudes and levels of education. Even if this selection is mainly due to being recruited through the university, if we consider that not all patients suffering from schizophrenia exhibit these aptitudes, this could constitute a bias in the selection of the sample.

Concerning the main paradigm ANT, Macleod et al. (2010)[32] found a low split-half reliability for the alerting network in healthy subjects. Nevertheless, we used here a modified version of ANT more reliable than the version elaborated by Fan et al. (2002)[26,36],. Another possible limitation of this work lies in the exploration of the "interaction" of attentional networks. The concept in itself involves the construction of a mixed block of trials, including warning, orienting and executive stimuli, with trials presumed to reflect the different combinations. Van der Lubbe et al. [52] showed that the use of a mixed block design provides an uncertainty about whether a warning signal, a cue or a target will appear first. This situation requires strong executive control to withhold automatic responses to warning signals, cues or incongruent targets. It creates a proactive inhibitory control that is released only when a target has been identified. Jaffard et al. (2007)[38] observed that the alerting effect as assessed with warning signals could be fully confounded with the behavioural outcomes of proactive inhibition control. Hence there could be confusion between an effect of an alerting cue reflecting a true phasic modulation of arousal, and an alerting effect relying more on an executive control mechanism. Boulinguez et al. (2009)[53] observed that the presentation of a warning signal involves an important executive control network inhibiting the mechanisms underlying movement initiation. A network of structures is implicated in the proactive inhibition mechanism with a strong involvement of the medial prefrontal cortex [54]. This point is of crucial interest in schizophrenia, where disorders of movement initiation and impairment of top-down inhibitory control have been found in orienting [55] as well as in oculomotor tasks, with frontal structures being strongly implicated [56]. All the studies demonstrating this proactive inhibition mechanism have been conducted with visual warning signals. We can speculate whether an auditory alerting signal also enhances a proactive inhibition mechanism. However, to precisely separate the effect of an interaction between alerting, orienting and executive attentional networks from an extended executive control effect by proactive inhibition, new study designs could include mixed block and pure warned/not warned block design tasks.

To conclude, the use of Callejas et al's version of ANT (2004)[32] gave us striking results showing the benefit of having clear measures for attentional networks and their interaction. We demonstrated that reinforcing an alerting state by a correct orientation can positively influence performance in difficult executive control conditions in schizophrenia. In other words, increasing orienting by alertness permits patients to better benefit from these cues in conflict situations. In addition, we found that, although the attentional networks have some functional independence, they modulate each other to bring about changes in adaptive behaviour and executive functions. This approach could provide fruitful ways of optimizing performance in schizophrenic patients in a multitude of cognitive domains.

## List of Abbreviations

ANT: Attentional Networks Test; C: healthy controls; PANSS: Positive and Negative Syndrome Scale; RT: Reaction Time; SZ: outpatients with schizophrenia; WAIS-R: Wechsler Adult Intelligent scale Revised version

## Competing interests

The authors declare that they have no competing interests.

## Authors' contributions

IA, SL, CM, DW participated in the experiment, the analysis and the writing of the manuscript

JL participated in the drawing of the experiment, the analyses and the writing of the manuscript

M-OK and J-PO participated in the writing of the manuscript.

All authors read and approved the final manuscript.
